# Vestibular and audiometric results after endolymphatic mastoid shunt surgery in patients with Menière’s disease

**DOI:** 10.1007/s00405-022-07582-6

**Published:** 2022-08-15

**Authors:** Jennifer L. Spiegel, Ivelina Stoycheva, Bernhard G. Weiss, Mattis Bertlich, Tobias Rader, Martin Canis, Friedrich Ihler

**Affiliations:** 1 Department for Otorhinolaryngology, University Hospital, Ludwig-Maximilians-Universität München, Marchioninistr. 15, 81377 Munich, Germany; 2 German Center for Vertigo and Balance Disorders, University Hospital, Ludwig-Maximilians-Universität München, Marchioninistr. 15, 81377 Munich, Germany; 3 Department of Ear, Nose, Throat, Head and Neck Surgery, Asklepios Kliniken Bad Tölz, Schützenstraße 15, 83646 Bad Tölz, Germany; 4 Department of Dermatology, University Hospital, Ludwig-Maximilians University of Munich, Munich, Germany

**Keywords:** Endolymphatic sac surgery, Endolymphatic mastoid shunt surgery, EMSS, Menière’s disease, MD

## Abstract

**Purpose:**

Treatment of Menière’s Disease (MD) comprises an array of both non-destructive and destructive treatment options. In patients who are therapy*–*refractory to non-destructive medical treatment, endolymphatic mastoid shunt surgery (EMSS) is both recommended and debated controversially. The aim of this study was to investigate safety in terms of hearing, vestibular function, complication rate, and efficacy with regards to vertigo control of EMSS in patients with MD according to the current diagnostic criteria of 2015.

**Methods:**

Retrospective analysis of 47 consecutive patients with definite or probable MD with description of demographic parameters, pre- and postoperative MD treatment, pre- and postoperative audiometric (pure tone audiometry) and vestibular (caloric testing) results. The parameters were compared between patients with and without postoperative vertigo control.

**Results:**

31/47 patients (66.0%) had improved vertigo control postoperatively. Postoperative hearing and vestibular preservation were predominantly stable. No significant differences between patients with improved vertigo control and patients with no change or worse vertigo episodes were found. In the treatment refractory group, 4 patients required a revision EMSS and 6 a destructive MD treatment (5 gentamicin intratympanically, 1 labyrinthectomy). No peri- or postsurgical complications were reported.

**Conclusions:**

EMSS was found to be beneficial in two thirds of the patients with definite or probable Morbus Menière and a safe procedure regarding hearing and vestibular preservation with no postoperative complications. Therefore, EMSS should be considered before inducing destructive treatment options, such as intratympanic gentamicin application or labyrinthectomy.

## Introduction

Meniere’s disease (MD) is a diverse disorder defined by three core symptoms: episodic vertigo, tinnitus, and sensorineural hearing loss. Both intrinsic, e.g., genetics, and extrinsic factors, e.g., influences from environment, may play a role in the development of MD [1]. The underlying pathomechanism of MD is well-discussed, though still unknown. Histopathological studies from the late 1980s hypothesize an association with an endolymph hydrops causing this fluctuating disease pattern [2]. The current diagnostic criteria published in 2015 define MD as a clinical syndrome characterized by recurrent, spontaneous vertigo attacks and fluctuating aural symptoms (sensorineural hearing loss, tinnitus, and aural fullness) [3]. MD patients suffer from an impaired quality of life [4, 5] and, therefore, require profound education about the available treatment options, which include both medical and surgical techniques, as well as non-destructive and destructive methods. Depending on the presented constellation of symptoms, the treatment concept should be tailored to the patient individually. An international team of MD experts of 4 different continents recommended the staged consideration of treatment options, based on a literature review and their own experiences [6] and presented an algorithm for the different forms of MD following five steps: the first step should consist of a conservative medical treatment including lifestyle adjustments, as in adequate sleeping [7], investigation of sleep apnea [8], decreased stress, avoidance of caffeine, alcohol, and tobacco, and adaption of a low salt diet. Vestibular rehabilitation and psychotherapy are recommended, as well [9–11]. In addition to those lifestyle changes, a medical treatment with betahistine can be initiated [12, 13]. As a second step and also non-destructive treatment, the intratympanic application of corticosteroids (dexamethasone or methylprednisolone) is recommended [14]. Since the treatment with intratympanic corticosteroid has shown satisfactory results, a decline in non-destructive surgical treatment with endolymphatic sac surgery has been observed. Therefore, it is recommended as a third-line treatment. It has long been criticized as a placebo-surgery, but a recent systematic review concluded low evident effect in improving symptoms in MD [15, 16]. Manipulation on the inner ear to decompress the endolymphatic sac evolved in the 1920s, when Portmann drew parallels between glaucoma [17] which was later expanded by William House in the 1960s by inserting material for a permanent shunt into the subarachnoid space or mastoid [18]. A recent review from Kersbergen and Ward thoroughly depicts the history regarding surgical manipulation of the inner ear treating MD [19]. Furthermore, four types of surgical techniques have been introduced, ranging from the most minimal invasive option, endolymphatic sac decompression, over endolymphatic sac incision, endolymphatic*–*mastoid shunt to the most invasive technique, the endolymphatic*–*subarachnoid shunt. The fourth step is a destructive medical technique with application of the ototoxic agent gentamicin intratympanically, which is stated as an effective method to eradicate vertigo in MD [20, 21]. The downside of this technique is the ototoxic effect in the cochlea that leads to permanent hearing impairment in some cases. As a fifth and final step the surgical destruction of the vestibular organ is seen as ultima ratio. Literature on vestibular neurectomy or labyrinthectomy is scarce. Older studies from the 1990s or 2000s revealed a more efficient treatment of vertigo attacks with vestibular neurectomy than with intratympanic gentamicin injection [22, 23]. Alongside with the labyrinthectomy a simultaneous cochlear implantation is recommended [6].

In the present study we focus on the well-debated efficacy of non-destructive endolymphatic sac surgery (endolymphatic mastoid shunt surgery—EMSS) in patients fulfilling the current diagnostic criteria for MD and investigating the preservation of postoperative hearing and vestibular function.

## Materials and methods

### Patient selection and ethical considerations

The retrospective data analysis was approved on April 11th 2019 by the Institutional Review Board of the University Hospital, LMU Munich (Ethikkommission der Medizinischen Fakultät der LMU München), reference number 19-086. Data collection was performed using the electronical clinical patient registry, including surgical reports, medical history, course of treatment, audiometric, and caloric testing pre- and postoperatively. Demographic data for each patient included sex, age, date of surgery, and date of last visit. Our study revealed 72 consecutive patients with MD at our academic tertiary referral center, who underwent EMSS between 2004 and 2019. A subset of 15 patients received EMSS and CI simultaneously as a first line surgical treatment and were not included to this study due to lack of follow-up of audiometric and caloric testing. Another 10 patients were excluded due to lack of data to categorize them along the diagnostic criteria of 2015 [3]. The remaining 47 patients with definitive or probable MD were divided into two groups: patients who reported of reduced or complete subsidence of vertigo attacks post-surgery, while patients in the second group had no benefit or worsening of symptoms after surgery.

### Surgical procedure—endolymphatic mastoid shunt surgery (Fig. 1)

**Fig. 1:**
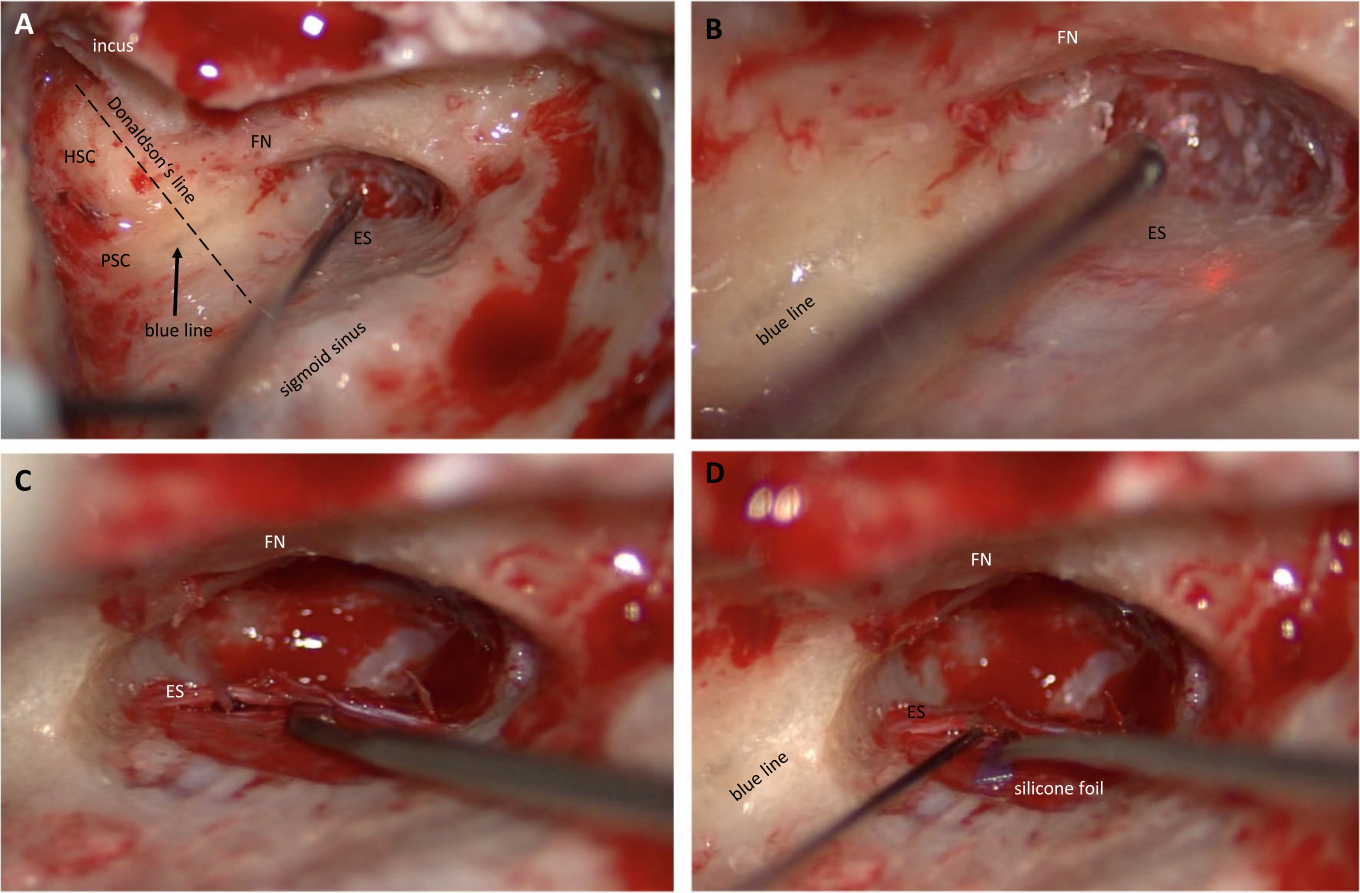
Endolymphatic*–*mastoid shunt surgery. Depiction of the endolymphatic*–*mastoid shunt surgery with A dissection of the landmarks: skull base to the middle fossa, sigmoid sinus, antrum with short process of the incus, facial nerve in the mastoid segment, horizontal, and posterior semicircular canal (“blue line”). The *Donaldson’s line* (dashed line) is drawn along the horizontal semicircular canal (HSC) and cuts the PSC perpendicularly. B Endolymphatic sac is identified lying inferior-posteriorly to the “blue line” of the posterior semicircular canal (PSC) as a duplicature of the dura mater (C) and incised. D Small triangular silicone foil (edge length 2 × 3 mm) is inserted as a permanent shunt. *ES* endolymphatic sac, *FN* facial nerve, *HSC* horizontal semicircular canal, *PSC* posterior semicircular canal

All patients received a standard EMSS procedure with application of a silicone foil as drainage. The procedure includes a mastoidectomy and dissecting the following landmarks: skull base to the middle fossa, sigmoid sinus, antrum with short process of the incus, facial nerve in the mastoid segment, horizontal, and posterior semicircular canal. The posterior semicircular canal was thinned out until the semicircular canal is visible as a blue line (Fig. 1A). The endolymphatic sac was identified lying inferior-posteriorly to the posterior semicircular canal as a duplicature of the dura mater. After thinning and removal of the bone superficial to the endolymphatic sac (= endolymphatic sac decompression; Fig. 1A, B), it was opened (Fig. 1C) and a small triangular silicone foil (edge length 2 × 3 mm) was inserted as a shunt (Fig. 1D). Postoperatively all patients received regular otologic follow-up treatment including audiometric testing of bone conduction, as well as monitoring for facial nerve impairment or nystagmus.

### Audiometry

Audiometric tests were performed as a pure tone audiometry with testing for each ear at 0.5, 1, 2, 3, 4, 6, and 8 kHz via headphone and with air and bone conduction thresholds between − 10 dB and 120 dB hearing level (dB HL) for each ear separately. Aided air conduction was measured with warble tones in free-field. Thresholds exceeding 120 dB HL were recorded as 130 dB HL for statistical purposes.

### Caloric testing

To evaluate the vestibular function, caloric testing on electronystagmography (ENG) was performed pre- and postoperatively using cold (30 °C) and warm (44 °C) water. To calculate for unilateral weakness the Jongkees-formula was applied [24].

### Data analysis

For preoperative analysis the worst audiometric result within 6 months prior to EMSS was considered. Regarding postoperative analysis, the first bone and air conduction audiometry after surgery, and for long-term results the last documented audiometric test was used. As per applicability to the study, this report was generated according to the *Strengthening the Reporting of Observational studies in Epidemiology statement* [25]. Summary data of audiometric tests was calculated according to the guidelines of the *Committee on Hearing and Equilibrium of the American Academy of Otolaryngology–Head and Neck Surgery* with the pure tone average for the air conducted frequencies 0.5, 1, 2, and 3 kHz (AC-PTA4^CHE^) [26]. In addition, bone conduction stability was analyzed with pure tone average for the bone conducted frequencies 0.5, 1, 2, and 4 kHz (BC-PTA).

### Statistical analysis

Statistical analysis was performed using SPSS (version 24) software (SPSS Inc, Chicago, IL). Shapiro*–*Walk test was used to test for normative distribution. Since the cohort is rather small, only descriptive statistical analysis was performed. All figures were created with Microsoft Excel version 1906.

## Results

### Demography and course of MD treatment

Of all included 47 patients, 31 (66.0%) reported of reduced or complete subsidence of vertigo attacks post-surgery. 16 patients (34.0%) accounted for the group of patients, who reported of no benefit or worsening of symptoms after surgery. No postsurgical complications (immediate postoperative vertigo, significant drop of bone conduction threshold, deafness, wound healing issues, postoperative infection, scarring issues) arose in both groups. The mean duration of first consultation until last follow-up was 4.6 ± 3.8 years and slightly shorter in the group with improved symptoms. The mean of age, sex, and side of the involved ear was evenly distributed in both groups. In the improved group 58.1% (*n* = 18) patients were diagnosed with definitive MD and 62.5% (*n* = 10) in the treatment refractory group. Regarding preoperative treatment, the majority of the patients (*n* = 38, 80.9%) in both groups were unsuccessfully treated with betahistine prior to EMSS. An ear tube was received by 25.5% (*n* = 12) of all patients. Intratympanic gentamicin injection was performed in 2 patients (4.3%) and intratympanic steroid injection in 2 patients of the improved group (6.5%).

Concerning postoperative treatment, the average postoperative follow-up was considerably longer in treatment refractory group (improved group: 15.9 ± 13.1 months vs. treatment refractory group: 23.5 ± 21.3 months). Despite reported improvement or subsidence of symptoms, a subset of 7 patients (22.5%) in the improved group continued with medication of betahistine. Due to lack of benefit or worsening of MD symptoms, 56.3% (*n* = 9) of the treatment refractory group received postoperative medical MD treatment. A destructive MD treatment was performed in 5 patients (31.3%) with 5 patients receiving gentamicin and 1 of those patients a labyrinthectomy. All data are depicted in Table 1.

**Table 1:** Patients’ characteristics

	Improved group	No benefit group	Total
Sex [*n* (%)]			
Female	13 (41.9)	10 (62.5)	23 (48.9)
Male	18 (58.1)	6 (37.5)	24 (51.1)
Age [years ± SD]	58.2 ± 12.2	50.9 ± 13.4	56.2 ± 13.0
Side [*n* (%)]			
Right	16 (51.6)	9 (56.3)	25 (53.2)
Left	19 (48.4)	7 (43.8)	26 (55.3)
Duration of symptoms [years ± SD (*n*)]	4.3 ± 3.4 (30)	4.8 ± 3.7 (14)	4.6 ± 3.8 (44)
Previous treatments [*n* (%)]			
Betahistine	27 (87.1)	11 (68.8)	38 (80.9)
IT gentamicin	1 (3.2)	1 (6.3)	2 (4.3)
Ear tube	6 (19.4)	6 (37.5)	12 (25.5)
IT dexamethasone	2 (6.5)	0 (0)	2 (4.3)
Disease category^a^ [*n* (%)]			
Definitive Morbus Menière	18 (58.1)	10 (62.5)	28 (59.6)
Probable Morbus Menière	13 (41.9)	6 (37.5)	19 (40.4)
Postoperative treatment			
No therapy [*n* (%)]	27 (77.4)	4 (25.0)	31 (66.0)
Second line therapy [*n* (%)]			
Betahistine	7 (22.5)	4 (25.0)	11 (23.4)
IT gentamicin	0 (0)	5 (31.3)	5 (10.6)
IT dexamethasone	0 (0)	1 (6.3)	1 (2.1)
Revision EMSS	0 (0)	4 (25.0)	4 (8.5)
Labyrinthectomy	0 (0)	1 (6.3)	1 (2.1)
Total [*n*]	31	16	47

Patient’s characteristics including pre- and postoperative additional Meniére specific treatment

*EMSS* endolymphatic sac surgery, *IT* intratympanic application, *n* number; *SD* standard deviation, *yr* years

^a^According to Lopez-Escamez et al. [3]

### Audiometric results

**Fig. 2:**
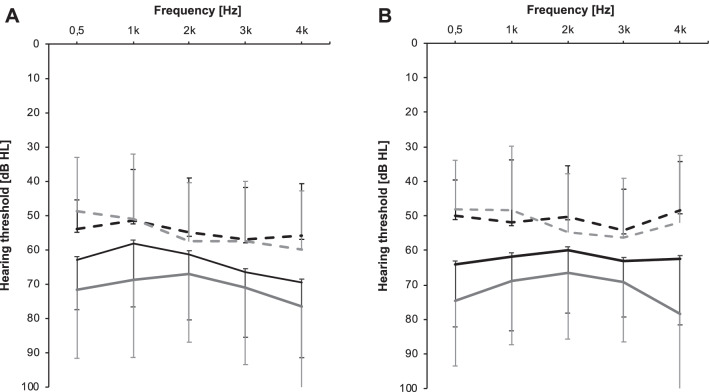
Pure tone audiometry pre- and postoperative. Pure tone audiometry pre- (black) and postoperatively (grey) in **A** all 31 patients who reported of better vertigo control after endolymphatic mastoid shunt surgery and **B** all 16 patients who showed no improvement after endolymphatic mastoid shunt surgery. Bone conduction is depicted as dotted lines and air conduction as solid lines. Standard deviation is indicated by whiskers

**Fig. 3:**
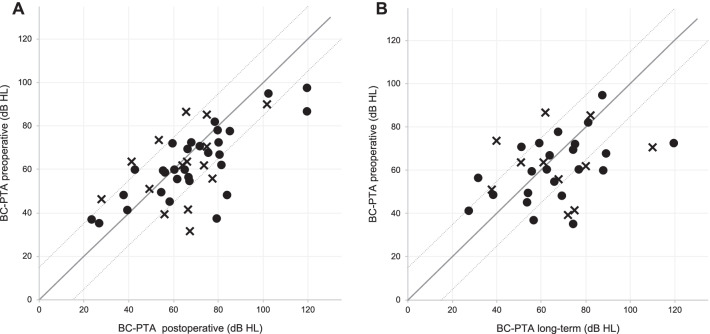
Scattergram of pre- vs. postoperative and preoperative vs. long-term bone conduction pure tone average. Change in bone conduction pure tone average of the frequencies 0.5, 1, 2, and 3 kHz of those patients who reported of better vertigo control after endolymphatic mastoid shunt surgery (marked as a circle) and those who showed no improvement after endolymphatic mastoid shunt surgery (marked as a cross). **A** Depicts the immediate postoperative results from 31 improved and 16 patients without benefit from the procedure; **B** shows the long-term results of 22 improved and 10 no-benefit patients. The solid line indicates no alterations; the area between the dotted lines indicates unaltered thresholds within a ± 15 dB HL range. BC: bone conduction; HL: hearing level; PTA^CHE 1995^: pure tone average as indicated by the *Committee on Hearing and Equilibrium* (CHE) [26]

All patients exhibited hearing impairment preoperatively according to WHO criteria (AC-PTA4^WHO^—air conduction of the frequencies 0.5, 1, 2, and 4 kHz) with no differences between both groups (Table 2). Bone conduction thresholds of the treated ears remained stable in both groups after surgery, air conduction seemed to have improved postoperatively (Fig. 2A, B). Postoperative bone conduction was stable in both groups as depicted by scattergrams (BC-PTA—bone conduction of the frequencies 0.5, 1, 2, and 4 kHz), with 87.1% (*n* = 27) in the improved group and 93.8% (*n* = 15) in the treatment refractory group exhibited preserved hearing (all 89.4%, *n* = 42). Long-term results were available in 21/31 of the improved patients and 9/16 treatment refractory patients, showing a slight decrease in hearing preservation (improved group: 90.5%, *n* = 19; treatment refractory group: 77.8%, *n* = 7; all 86.7%, *n* = 26; Fig. 3A, B).

**Table 2:** Hearing loss

	Improved group	No benefit group	Total
Follow-up audiogram preoperative (months ± SD)	1.5 ± 1.5	1.2 ± 0.9	1.4 ± 1.2
AC-PTA of hearing loss preoperative (dB ± SD)	63.7 ± 18.6	62.3 ± 18.6	63.0 ± 18.6
Grade of hearing loss preoperative [*n* (%)]^a^	*n* = 31	*n* = 16	*n* = 47
No impairment	0 (0)	0 (0)	0 (0)
Slight impairment	2 (6.5)	1 (6.3)	3 (6.4)
Moderate impairment	14 (45.2)	5 (31.3)	19 (40.4)
Severe impairment	11 (35.5)	3 (18.8)	14 (29.8)
Profound impairment or deafness	4 (12.9)	7 (43.8)	11 (23.4)
Follow-up audiogram postoperative (months ± SD)	1.6 ± 2.3	1.3 ± 1.5	1.5 ± 1.9
AC-PTA of hearing loss postoperative (dB ± SD)	70.9 ± 23.7	65.4 ± 19.4	68.2 ± 21.5
Grade of hearing loss postoperative [*n* (%)]^a^	*n* = 31	*n* = 15	*n* = 46
No impairment	0 (0)	0 (0)	0 (0)
Slight impairment	4 (10.8)	1 (6.6)	5 (10.9)
Moderate impairment	4 (16.2)	4 (26.7)	8 (17.4)
Severe impairment	12 (32.4)	7 (46.7)	19 (41.3)
Profound impairment or deafness	11 (40.5)	3 (20.0)	14 (30.4)
Follow-up audiogram postoperative, long-term (months ± SD)	15.8 ± 13.1	23.5 ± 21.3	19.7 ± 17.2
AC-PTA of hearing loss postoperative, long-term (dB ± SD)	68.3 ± 21.4	71.6 ± 19.1	70.0 ± 20.3
Grade of hearing loss postoperative, long-term [*n* (%)]^a^	*n* = 22	*n* = 10	*n* = 32
No impairment	0 (0)	0 (0)	0 (0)
Slight impairment	3 (10.7)	1 (10.0)	4 (12.5)
Moderate impairment	7 (21.6)	1 (10.0)	8 (25.0)
Severe impairment	7 (32.1)	5 (50.0)	12 (37.5)
Profound impairment or deafness	5 (21.6)	3 (30.0)	8 (25.0)

Audiometry results with the grade of hearing loss in dB hearing level including the time of testing

*n* number, *AC-PTA* pure tone average (air conduction frequencies: 0,5, 1, 2, 3, and 4 kHz)

^a^According to WHO

### Vestibular function

Of all 47 patients in a subset of 22 patient both pre- and postoperative caloric testing results were available for analysis (improved group: 15/31 patients; treatment refractory group: 7/16 patients). The postoperative ENG was performed within 13.8 ± 11.7 months after surgery. Pre- vs. postoperative caloric testing results showed comparable values in both groups. In the improved group 5 patients (33.3%, 5/15) changed from a normal to an abnormal response in caloric testing on the operated side. In the treatment refractory group, 1 patient (14.3%, 1/7) had an abnormal result postoperatively. The mean peripheral vestibular response to caloric stimulation was stable in both groups. In none of the patients a complete loss of vestibular function was observed (Table 3).

**Table 3:** Caloric testing of vestibular function

	Improved group	No benefit group	Total
Mean time preoperative [months ± SD]	6.2 ± 6.7	5.0 ± 5.5	5.8 ± 6.4
Mean follow-up postoperative [months ± SD]	13.8 ± 11.7	13.7 ± 13.6	13.8 ± 12.6
Abnormal [*n* (%)]			
Preoperative	4 (26.7)	2 (28.6)	6 (27.3)
Postoperative	7 (46.7)	2 (28.6)	9 (40.9)
Change in pre-/postoperative vestibular excitability [*n* (%)]			
Remained normal	6 (40.0)	4 (57.1)	10 (45.5)
Abnormal/normal	2 (13.3)	1 (14.3)	3 (13.6)
Remained abnormal	2 (13.3)	1 (14.3)	3 (13.6)
Normal/abnormal	5 (33.3)	1 (14.3)	6 (27.3)
Mean peripheral vestibular excitability [% ± SD]			
Preoperative	46.2 ± 20.5	37.7 ± 32.4	40.7 ± 24.3
Postoperative	43.1 ± 22.8	36.0 ± 31.1	40.0 ± 25.0
Mean vestibular excitability [°/s ± SD]			
Preoperative	10.7 ± 5.6	11.6 ± 9.9	11.0 ± 7.0
Postoperative	7.7 ± 4.4	10.2 ± 6.5	8.5 ± 5.1
Number of patients whose mean peripheral vestibular excitability [*n* (%)]			
Decreased > 10%	2 (13.3)	1 (14.3)	3 (13.6)
Decreased > 20%	2 (13.3)	1 (14.3)	3 (13.6)
Increased > 25%	3 (20.0)	1 (14.3)	4 (18.2)
Total (*n*)	15	7	22

Caloric testing of periphery vestibular function including time of testing

*EMSS* endolymphatic sac surgery, *mo* months, *n* number, *RVR* reduced vestibular response, *SD* standard deviation

## Discussion

The efficacy of endolymphatic sac surgery is well-debated and literature on it is broad (Table 4). The surgical techniques differ and range from a simple decompression of the endolymphatic sac to the invasive procedure of creating a subarachnoidal shunt. The most common technique reported in the literature is endolymphatic mastoid shunt surgery (EMSS) which can be performed with the mere incision of the endolymphatic sac or with creating a permanent shunt by applying a small silicone foil to the incision, which was performed in the study at hand.

**Table 4:** Literature on endolymphatic sac surgery

Year	Author	No. of patients	Form of endolymphatic sac surgery	Follow-up	Vertigo control	Hearing preservation	Version of diagnostic criteria	Audiometic results (stage of MD/PTA)	Preoperative audiometric testing	Preoperative vestibular testing	Postoperative audiometric testing	Postoperative vestibular testing	Vestibular migraine/other
1977	Arendberg et al. [38]	35	EMSS with silicone	n.g	88.9%	85.2%	1985	Y	N	N	N	N	n.g./n.g
1979	Arendberg et al. [39]	66	EMSS with silicone	10.3 mo	90.8%	n.g	1985	Y	N	N	N	N	n.g./n.g
1981	Thomsen et al. [32]	15	EMSS with silicone	12 mo	87%	87%	1972	Y	N	N	N	N	n.g./Y
1983	Brown et al. [40]	245/328	EMSS vs. ES decompression	n.g	n.g	n.g	n.g	N	n.g	n.g	n.g	n.g	n.g./n.g
1983	Goldenberg et al. [41]	48	EMSS with silicone	1–5 yrs	81%	n.g	n.g	N	n.g	n.g	n.g	n.g	n.g./n.g
1983	Miller et al. [42]	24	EMSS with silicone	> 12 mo	87%	79%	n.g	Y	N	n.g	N	n.g	n.g./n.g
1983	Spector et al. [43]	122	EMSS with silicone	3 yrs	83%	63%	1972	Y	N	Y	N	Y	n.g./n.g
1983	Thomsen et al. [33]	13	EMSS with silicone	36 mo	69%	69%	1972	Y	N	N	N	N	n.g./Y
1984	Bretlau et al. [36]	13 vs. placebo	EMSS	36 mo	70%	61%	1972	Y	N	N	N	N	n.g./n.g
1984	Glasscock et al. [44]	310	ES subarachnoidal shunt	1–11 yrs	66%	65%	1972	N	N	N	N	N	n.g./n.g
			EMSS	1–11 yrs	50%	57%	1972	N	N	N	N	N	n.g./n.g
			Arendberg valve procedure	1–11 yrs	49%	53%	1972	N	N	N	N	N	n.g./n.g
1985	Huang et al. [45]	339	EMSS with/ without silicone	n.g	80%	n.g	n.g	Y	N	n.g	N	n.g	n.g./n.g
1986	Smyth et al. [46]	21	ES decompression	12 mo	86%	86%	n.g	Y	N	N	N	N	n.g./Y
		21	ES decompression	8–10 yrs	71%	52%	n.g	Y	N	N	N	N	n.g./Y
1986	Thomsen et al. [34]	12	EMSS with silicone	84 mo	75%	42%	1972	Y	N	N	N	N	n.g./Y
1987	Arendberg et al. [47]	214	EMSS with silicone	35.1 mo	73.9%	60.3% worse	1985	n/a	Y	N	Y	N	n.g./n.g
1987	Brackmann et al. [48]	169	EMSS with silicone	> 12 mo	71.1%	62.2%	1985	N	N	N	N	N	n.g./n.g
1987	Kitahara et al. [49]	140	EMSS without silicone	24 mo	94%	60.7%	1985	Y	N	N	N	N	n.g./n.g
1988	Luetje et al. [50]	29/189	ES subarachnoid shunt	< 24 mo	76%	74%	1972	Y	N	N	N	N	n.g./n.g
		4/189	ES tube	< 24 mo	75%	95%	1972	Y	N	N	N	N	n.g./n.g
		6/189	EMSS	< 24 mo	83%	95%	1972	Y	N	N	N	N	n.g./n.g
		10/189	ES marsupialization	< 24 mo	80%	95%	1972	Y	N	N	N	N	n.g./n.g
		4/189	EMSS with silicone	< 24 mo	100%	95%	1972	Y	N	N	N	N	n.g./n.g
		68/189	ES subarachnoid shunt	> 24 mo	91%	67%	1972	Y	N	N	N	N	n.g./n.g
		48/189	ES tube	> 24 mo	87%	73%	1972	Y	N	N	N	N	n.g./n.g
		6/189	EMSS	> 24 mo	83%	80%	1972	Y	N	N	N	N	n.g./n.g
		3/189	ES marsupialization	> 24 mo	33%	100%	1972	Y	N	N	N	N	n.g./n.g
		3/189	EMSS with silicone	> 24 mo	100%	100%	1972	Y	N	N	N	N	n.g./n.g
1988	Monsell et al. [51]	63	EMSS with silicone	24 mo	90%	41%	1972/1985	Y	N	N	N	N	n.g./n.g
1989	Bretlau et al. [52]	11 vs. placebo	EMSS	108 mo	90.9%	n.g	1972	Y	N	N	N	N	n.g./n.g
1993	Telischi et al. [53]	234	ES subarachnoidal shunt	> 10 yrs	63%	n.g	n.g	N	N	N	N	N	n.g./n.g
1994	Moffat et al. [54]	100	EMSS	n.g	81%	74%	1985	Y	N	N	N	N	n.g./Y
1996	Welling et al. [55]	10	EMSS with silicone	> 24 mo	60%	66%	1985	Y	N	N	N	N	n.g./n.g
1997	Quaranta et al. [56]	20	EMSS with silicone	> 5 yrs	85%	65%	n.g	Y	N	N	N	N	n.g./n.g
1998	Gianoli et al. [57]	37	ES decompression	12 mo	85%	86%	1985	Y	N	Y	N	N	n.g./Y
				24 mo	100%	85%	1985	Y	N	Y	N	N	n.g./Y
1998	Pensak et al. [58]	96	EMSS with silicone	5 yrs	91%	n.g	1995	N	N	N	N	N	n.g./n.g
1998	Quaranta et al. [59]	20	EMSS	6 yrs	85%	45%	1985	Y	N	Y	N	N	n.g./n.g
1998	Sajjadi et al. [60]	27	ES decompression	1–7 yrs	85%	70%	1972	Y	N	N	N	N	n.g./Y
1998	Thomsen et al. [61]	15	EMSS with silicone	No change 6 mo–12 mo	86%	86%	1995	Y	N	N	N	N	n.g./Y
1999	Huang et al. [62]	51	EMSS with silicone	n.g	94.1%	88,20%	1985	Y	N	N	N	N	n.g./n.g
2001	Quaranta et al. [63]	15/45	ES decompression	n.g	n.g	67%	n.g	Y	N	N	N	N	n.g./n.g
		15/45	EMSS with silicone	n.g	n.g	87%	n.g	Y	N	N	N	N	n.g./n.g
2001	Sennaroglu et al. [64]	25	ES decompression	n.g	52%	72%	1985	Y	N	n.g	N	n.g	n.g./Y
2003	Ostrowski et al. [65]	68	ES decompression	55 mo	72%	82%	1995	Y	N	Y	N	N	n.g./Y
2005	Kaylie et al. [66]	74	EMSS	18–24 mo	72,8	72%	1995	Y	N	N	N	N	n.g./n.g
2006	Convert et al. [67]	21/59	EMSS with silicone	24 mo	71.9%	71.1%	1995	Y	N	Y	N	n.g	n.g./Y
		38/59	ES decompression	24 mo	71.9%	71.1%	1995	Y	N	Y	N	n.g	n.g./Y
2007	Brinson et al. [68]	108/196	ES decompression	18–24 mo	75%	54%	1995	Y	N	n.g	N	n.g	n.g./Y
		88/196	EMSS with silicone	18–24 mo	75%	62%	1995	Y	N	n.g	N	n.g	n.g./Y
2008	Lee et al. [69]	226	EMSS	> 15 yrs	87%	n.g	1995	N	N	n.g	N	n.g	n.g./n.g
2008	Wetmore et al. [70]	51	EMSS with silicone	n.g	78%	n.g	1995	Y	N	N	N	N	n.g./n.g
2010	Derebery et al. [71]	183	EMSS with silicone	12–24 mo	86%	71%	1995	Y	N	n.g	N	n.g	n.g./n.g
2012	Kim et al. [37]	16	EMSS with silicone	1–18 mo	94%	100%	1995	Y	N	Y	N	Y	Y/Y
2017	Wick et al. [72]	6/53	EMSS with silicone + without steroid	90 mo (35–112)	66%	n.g	1995	Y	Y	N	Y	N	n.g./n.g
		20/53	EMSS with silicone + steroids intratympanic	90 mo (35–112)	83%	n.g	1995	Y	Y	N	Y	N	n.g./n.g
		27/53	EMSS with silicone + steroids intratympanic + intravenous	90 mo (35–112)	66%	n.g	1995	Y	Y	N	Y	N	n.g./n.g
2021	Gendre et al. [27]	73	ES decompression/EMSS	> 2 mo	67% (50% vs. 75%)	n.g	1995/2015	Y	Y	Y	Y	Y	n.g./n.g

Caloric testing of periphery vestibular function including time of testing

*EMSS* endolymphatic sac surgery, *ES* endolymphatic sac, *MD* Menière’s disease, *mo* months, *N* no, *n.g.* not given, *no.* number, *PTA* pure tone average, *vs.* versus, *Y* yes, *yrs* years

The study at hand shows that EMSS is a safe and non-destructive method, that results in improved symptom control in a fair share—around 2 thirds—of patients with MD, when other non-destructive methods such as betahistine or intratympanic application of corticosteroids failed. Not a single patient in the reported cohort exhibited postoperative complications and bone conduction thresholds remained stable. To our knowledge, this is the second study investigating the effect of endolymphatic sac surgery on patients diagnosed with MD according to the current diagnostic criteria from 2015 [27]. All patients received a detailed history and diagnostic workup ruling out vestibular migraine, vestibular neurinoma, vestibular paroxysm, autoimmune inner ear disorder, and other differential diagnoses. All other available studies in the literature applied older versions of the diagnostic criteria and information on differential diagnosis seem to be lacking (Table 4). Moreover, the study at hand depicts detailed pre- and postoperative audiometric and vestibular results showing the safety of this non-destructive treatment option for MD patients. All patients received the same type of endolymphatic sac surgery—a endolymphatic mastoid shunt with application of a triangular silicone foil which seems to be the most common technique according to the literature [28–30] (Table 4). The most crucial novel aspect of the study at hand is the comparison of results between both study subgroups: patients who seem to have benefitted from the procedure and those who have not. The focus has never been laid on categorizing patients regarding the treatment effect before. However, this might be essential to further investigate the disease and how those patients can be effectively treated. Comparing those two patient groups regarding demographic characteristics, distribution of gender and side, age, and disease category according to the current diagnostic criteria were similar. In addition, with regard to hearing, the results between both patient groups were similar, as well as results from peripheral vestibular testing with regard to pre- and postoperative values. Interestingly, patients who benefitted from the procedure, had slightly better values regarding the peripheral vestibular function (side difference around 10%) than those who did not benefit from the procedure, which might illustrate the potential stop of the disease progress by performing the EMSS. Nevertheless, the grade of vestibular excitability was similar. Due to the small sub cohort numbers (group 1: *n* = 15; group 2: *n* = 7) no sound conclusions can be drawn and analysis was solely descriptive. Therefore, larger numbers and a prospective study setting would be necessary to find correlations or even predicators to estimate preoperatively a treatment success. The limitations of the study lie in the retrospective nature of the study: the cohort is relatively small and results regarding vestibular excitability was only available in 22 patients. In addition, due to the retrospective character of the study, thorough documentation to categorize the patients on the basis of the current diagnostic criteria for MD was not sufficient in 10 patients who were excluded prior to analysis. Another limitation is the relatively short follow-up results. Most of the other studies in the literature have longer follow-up with up to 9 years (Table 4).

Investigations on the effect of endolymphatic sac surgery go back to the 1950s. To date, about 40 studies assessed endolymphatic sac surgery, predominately within a retrospective setting [31] (Table 4). The without doubt most striking study on endolymphatic sac surgery comes from Denmark and was conducted as a double-blind prospective study comparing the efficacy between EMSS with a silicone shunt and mastoidectomy as placebo surgery [32]. This group has published recurrently multiple follow-up results up to 9 years on their study and showed vertigo control rates between 69 and 87% with no significant difference between both study and placebo group [32–36]. However, the authors did not specify the extent of the mastoidectomy. Therefore, we do not know, how extensive the mastoidectomies were performed, which could have the same effect as an endolymphatic decompression surgery. However, since publication of these results, the efficacy of endolymphatic sac surgery has been called into doubt repeatedly and its benefit is still debated [31]. Nevertheless, vertigo control rate was reported in the literature between 49 and 100% (Table 4). Whether this effect indicates the genuine efficacy of EMSS or is solely accounted for a placebo effect [32–36] or even due to the characteristic phenomenon, that MD patients consult their physician at the climax of symptom severity, can only be answered by future blinded prospective placebo-controlled studies.

All in all, the follow-up duration of the study at hand (19.7 ± 17.2 months) is on the lower range of reports from the literature, where follow-up results from 12 months to 9 years are given (Table 4). Gender was similarly distributed as in most studies [27–30]. More than half of the studies focused on hearing preservation, as well, without presenting detailed information on the audiograms of the investigated cohort. Hearing preservation was very satisfactory postoperatively in the study at hand (80.9%), also with similar long-term results (68.8%) compared to studies in the literature, ranging from 41 to 100% (Table 4), which underlines the safety and non-destructive character of this MD treatment option. Decreased long-term hearing results might be influenced by patients with a therapy*–*refractory progressive MD and resulting hearing impairment. This can also be observed in the difference of results between the both groups. Only two other recent publications presented pre- and postoperative results from caloric testing. Kim et al. investigated the peripheral vestibular function via caloric testing and found no significant of changes pre- to postoperative values [37], as well as the researchers around Gendre et al. who in addition observed no postoperative value changes in vestibular evoked myogenic potentials and video head impulse test [27].

## Conclusions

The treatment effect of EMSS was found beneficial in two thirds of the patients with definite or probable MD. It is a safe procedure regarding hearing and vestibular preservation with a low complication rate. Therefore, EMSS should be considered before inducing destructive treatment options, such as intratympanic gentamicin application or labyrinthectomy. Regarding the classification of patients who benefit from EMSS and those who do not, no sound correlation with clinical or functional parameters were found. For further insights prospective studies with larger power are needed.

## Data Availability

Original data are available on demand.

## Code availability

Statistical analysis was performed using SPSS (version 24) software (SPSS Inc, Chicago, IL). Shapiro*–*Walk test was used to test for normative distribution. All figures were created with Microsoft Excel version 1906.
